# IL-2 Restores T-Cell Dysfunction Induced by Persistent *Mycobacterium tuberculosis* Antigen Stimulation

**DOI:** 10.3389/fimmu.2019.02350

**Published:** 2019-10-02

**Authors:** Xun Liu, Fei Li, Hongxia Niu, Lan Ma, Jianzhu Chen, Ying Zhang, Liang Peng, Chao Gan, Xingming Ma, Bingdong Zhu

**Affiliations:** ^1^Gansu Provincial Key Laboratory of Evidence Based Medicine and Clinical Translation and Lanzhou Center for Tuberculosis Research, School of Basic Medical Sciences, Lanzhou University, Lanzhou, China; ^2^School of Basic Medical Sciences, Institute of Pathogen Biology, Lanzhou University, Lanzhou, China; ^3^Department of Biology, Koch Institute for Integrative Cancer Research, Massachusetts Institute of Technology, Cambridge, MA, United States; ^4^Department of Molecular Microbiology and Immunology, Bloomberg School of Public Health, Johns Hopkins University, Baltimore, MD, United States; ^5^Center of Life Science, School of Life Sciences, Lanzhou University, Lanzhou, China

**Keywords:** *Mycobacterium tuberculosis*, T cell exhaustion, T cell dysfunction, antigen persistence, IL-2

## Abstract

Tuberculosis (TB) is a chronic disease mainly caused by *Mycobacterium tuberculosis*. The function of T cells usually decreased and even exhausted in severe TB such as multiple drug resistant TB (MDR-TB), which might lead to the failure of treatment in return. The mechanism of T cell dysfunction in TB is still not clear. In this study we set up a mouse model of T cell dysfunction by persistent *M. tuberculosis* antigen stimulation and investigated the therapeutic role of interleukin 2 (IL-2) in it. C57BL/6 mice were primed with *Mycobacterium bovis* Bacillus Calmette-Guérin (BCG) and boosted repeatedly with a combination of *M. tuberculosis* fusion proteins Mtb10.4-HspX (MH) plus ESAT6-Ag85B-MPT64_ <190−198>_-Mtb8.4-Rv2626c (LT70) or MH plus ESAT6 and CFP10 with adjuvant of N, N′-dimethyl-N, N′-dioctadecylammonium bromide (DDA) plus polyinosinic-polycytidylic acid (Poly I:C). Following persistent antigen stimulation, the mice were treated with IL-2 and the therapeutic effects were analyzed. The results showed that compared with the mice that received transient antigen stimulation (boost twice), persistent antigen stimulation (boost more than 10 times) resulted in decrease of antigen specific IFN-γ and IL-2 production, reduction of memory CD8^+^ T cells, over-expression of immune checkpoint programmed cell death protein 1 (PD-1), and impaired the protective immunity against bacterial challenge. Treating the T cell functionally exhausted mice with IL-2 restored antigen-specific T cell responses and protective efficacy. In conclusion, persistent stimulation with *M. tuberculosis* antigens induced T cell dysfunction, which could be restored by complement of IL-2.

## Introduction

Tuberculosis (TB) is a typical chronic infectious disease caused by *Mycobacterium tuberculosis*, which activates protective cell-mediated immune responses (1). However, the protective immunity declines at late stage of TB, and the TB patients would die of consumption eventually. The number of Th1-type CD4^+^ T cells declined in TB patients (2). CD4^+^ and CD8^+^ T cells were found functionally exhausted in mice infected with *M. tuberculosis* (3). We and other groups observed that T cells experienced dysfunction/exhaustion in severe miliary sputum positive cavitary tuberculosis and MDR-TB (4, 5). We suppose that T cells get functionally exhausted in sputum positive cavitary tuberculosis due to persistent stimulation by a large of bacteria proliferating in necrotic liquefied material inside cavitary lesions. Here, we set up a mouse model to investigate our prediction.

T-cell exhaustion was primarily identified in lymphocytic choriomeningitis virus (LCMV) infection (6), and also in cancers and other chronic viral infections such as human immunodeficiency virus (HIV), hepatitis B virus (HBV) and hepatitis C virus (HCV) (7–9). T-cell exhaustion is a process in which T cells lose their function progressively (10), with losing cytotoxicity and decreasing proliferation and IL-2 secretion first, followed by loss of IFN-γ and TNF-α production (11–13). The step-wise impairment of effector functions of antigen-specific T cell response will ultimately affect the host's ability to confer protection.

Some inhibitory receptors, such as PD-1, lymphocyte activation gene 3 (LAG-3), T cell immunoglobulin mucin 3 (TIM-3), cytotoxic T-lymphocyte-associated protein 4 (CTLA-4), are highly expressed on exhausted T cells during chronic viral infection and tumor progression (14–16). Up-regulation of PD-1 involved in various chronic viral infectious diseases such as HIV, HBV, HCV, and LCMV infection (17, 18), and blocking this pathway can rejuvenate CD8^+^ T cell function and enhance viral control (19). PD-1 (20) and TIM3 were found highly expressed in exhausted T cells in TB (3).

IL-2 is the most important cytokine that regulates the differentiation of T cells. IL-2 promotes the formation of effector CD8 T cells (21). Low-dose IL-2 favors generation of memory T cells (22, 23) and enhances CD8^+^ T cell responses in virus chronically infected mice by decreasing inhibitory receptor levels and increasing memory T cells–associated molecules CD127 and CD44 (24). IL-2 has been applied for activation and expansion of T cells *in vitro*. Adding IL-2 can overcome T cell anergy and recover T cells proliferation (25, 26). Moreover, IL-2 is proven to be effective in treating MDR-TB (27, 28) while its therapeutic results varies in common TB patients (29).

It is still unclear what causes T cell dysfunction/exhaustion in TB. We hypothesize that continuous antigen stimulation might be the direct reason to cause immune dysfunction in TB. To examine the hypothesis, mice were primed with BCG and boosted with a combination of *M. tuberculosis* antigens MH (Mtb10.4-HspX) (30) plus LT70 (ESAT6-Ag85B-MPT64_ <190−198>_-Mtb8.4-Rv2626c) (31) or MH plus ESAT6 and CFP10 weekly for more than 10 weeks to mimic persistent antigen stimulation as in severe *M. tuberculosis* infection. Then, we analyzed the function of T cells to investigate whether T-cell get functionally exhausted. In addition, IL-2 was used to treat persistent antigen–stimulated mice and the therapeutic effects of IL-2 were analyzed. We found that following persistent *M. tuberculosis* antigen stimulation, T cells got functionally exhausted, while complementing IL-2 could restore dysfunction and reinvigorate immunity.

## Materials and Methods

### Ethics Statement

All animal experiments were carried out under the guidelines of Council on Animal Care and Use, and the protocols were reviewed and approved by Institutional Animal Care and Use Committee of Lanzhou University. Animals were monitored daily and received free access to water and food throughout the study.

### Antigens Preparation

Antigens were prepared as previously described (30, 31). The *M. tuberculosis* fusion protein MH without affinity tag (30) was highly expressed in the supernatant of the recombinant *E. coli* strain BL21 lysate and successfully purified by chromatography. All column chromatography procedures including the initial ion-exchange chromatography (IEX) on Q-sepharose high performance column, hydrophobic chromatography (HIC) on butyl-sepharose high performance column and gel filtration chromatography (GFC) on Superdex 75 prep grade column were performed with AKTA Purifier 100 (GE Healthcare, Piscataway, NJ).

The method for purification of LT70 without affinity tag (31) included salting-out and HIC on butyl-sepharose high performance column, which was also carried out with AKTA Purifier 100 (GE Healthcare, Piscataway, NJ). The proteins ESAT6 and CFP10 with his-tag (32) was stably produced in the supernatant of recombinant BL21 lysate and eluted at 150 mM imidazole by Nickel Affinity Gel Column Chromatography.

### Schedules of *M. tuberculosis* Antigen Stimulation and IL-2 Treatment

Specific pathogen free C57BL/6 female mice (6–8 weeks old) (Gansu University of Traditional Medicine, Gansu, China) were primed with BCG (Shanghai strain, D2-PB302, a derivative of Copenhagen strain, provided by Lanzhou Institute of Biological Products) at a dose of 5 × 10^6^ bacterial colony forming units (CFU) once via subcutaneous administration and boosted with antigens. Two combinations of antigens were applied to boost BCG in different schedules.

**Figure 1:**
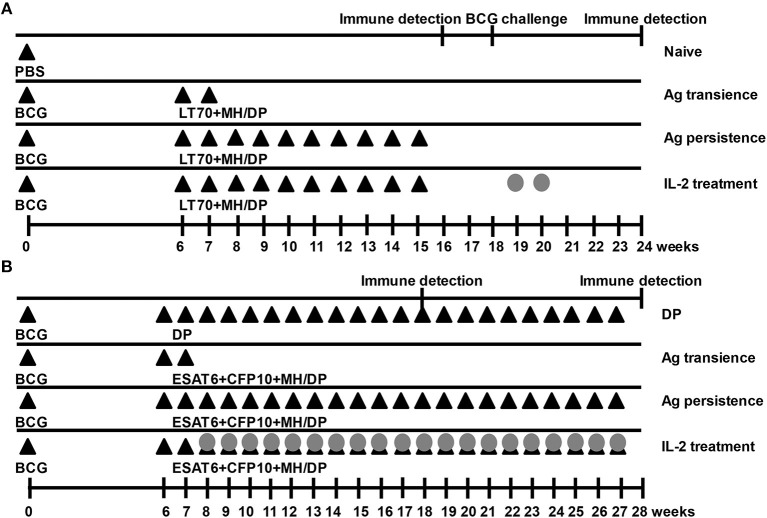
Immunization schedules. Mice were primed with BCG once and boosted with *M. tuberculosis* antigens (Ag) MH plus LT70 (ML for short) or MH plus ESAT6, CFP10 (MEC for short) weekly. The mice with persistent antigen stimulation were treated with IL-2. Then, the function of T cells was evaluated. **(A)** Immunization schedule with antigens ML. **(B)** Immunization schedule with antigens MEC. The adjuvant DDA plus Poly I:C (DP) or PBS were used as the sham control; Antigen transience, mice were primed with BCG once and boosted with the antigens only at 6th and 7th weeks; Ag persistence, boosting BCG with the antigens weekly for many weeks; IL-2 treatment, using IL-2 to treat persistent antigens–stimulated mice. DP, DDA plus Poly I:C; Closed triangles, antigen immune; Gray circles, IL-2 treatment.

In the first schedule (Figure 1A), 6 weeks after BCG priming, mice were boosted with a combination of *M. tuberculosis* antigens including 10 μg of MH plus 10 μg of LT70 (31) with the adjuvant consisting of 250 μg of DDA (Anhui Super chemical technology Co., Ltd., China) and 50 μg of Poly I:C (Kaiping Genuine Biochemical Pharmaceutical Co., Ltd., Guangdong, China) (33) via subcutaneous administration every 7 days for 10 weeks (Ag persistence group). Three weeks later, the function of T cells was assayed. From 18th week, the mice with persistent antigen stimulation were treated with 5 × 10^3^ IU IL-2 (Recombinant Human Interleukin-2 Proleukin®, 3SBio Group, Shenyang, China) in 100 μl via intraperitoneal injection daily for 2 weeks (IL-2 treatment group). Then, all groups of mice were challenged with BCG 1 week after IL-2 treatment. Three weeks later, the immune responses were evaluated. Boosting BCG with the antigens only at 6th and 7th weeks was used as a control of transient antigen stimulation (Ag transience group). The PBS was used as the sham control (Naive group).

In the second experiment (Figure 1B), 6 weeks after BCG priming, mice were boosted with a combination of *M. tuberculosis* antigens 5 μg of ESAT6, 5 μg of CFP10, and 10 μg of MH (30) with the adjuvant consisting of 250 μg of DDA (Anhui Super chemical technology Co., Ltd., China) plus 50 μg of Poly I:C (Kaiping Genuine Biochemical Pharmaceutical Co., Ltd., Guangdong, China) (33) via subcutaneous administration weekly for 22 weeks until T cells were proven to be functionally exhausted (Ag persistence group). Subsequently, the mice with persistent antigen stimulation were treated with 5 × 10^3^ IU IL-2 (Recombinant Human Interleukin-2 Proleukin®, 3SBio Group, Shenyang, China) in 100 μl *via* intraperitoneal injection daily, starting from 8th week daily for 20 weeks (IL-2 treatment group). One week after IL-2 treatment, the immune responses were evaluated. Boosting BCG with the antigens only at 6th and 7th weeks was used as a control of transient antigen stimulation (Ag transience group). The adjuvant of DDA plus Poly I:C was used as the sham control (DP group).

### Enzyme-Linked Immunospot Assay (ELISPOT) for IFN-γ Production in the Spleen

Lymphocytes were freshly isolated by gradient centrifugation from spleens. Mouse IFN-γ pre-coated ELISPOT kit (Dakewe Biotech Company Ltd., Shenzhen, China) was performed according to the instruction of the manual (30, 34). The 96-well plates pre-coated with anti-mouse IFN-γ antibody were activated with RPMI-1640 for 15 min at room temperature. Lymphocytes were plated in duplicate at 5 × 10^5^ cells per well in RPMI-1640 supplemented with penicillin, streptomycin, and 10% newborn calf serum (Genetimes Technology, Inc., Shanghai, China) and stimulated with Ag85B (35) (5 μg/ml) or purified protein derivative (PPD) (5 μg/ml), respectively, for 20 h at 37°C, 5% CO_2_. The cells were then removed and the biotinylated antibody, streptavidin-HRP and AEC substrate were added subsequently, spots were counted by an ELISPOT reader (Bio-sys, GmbH, Karben, Germany) and results were expressed as the mean of the spot forming cells (SCFs) per 5 × 10^5^ cells ± standard error of mean (SEM).

### Enzyme-Linked Immunosorbent Assay (ELISA) for IL-2 Secretion

Lymphocytes isolated from spleen cells were plated in 24-well plates at 5 × 10^6^ cell per well in RPMI-1640 supplemented with penicillin, streptomycin, and 10% newborn calf-serum and stimulated *ex vivo* with PPD (5 μg/ml) and Ag85B (5 μg/ml) for 4 h at 37°C for 3 days and the concentrations of IL-2 in culture supernatant were detected by ELISA according to the manufacturer's protocol (Mouse IL-2 ELISA kits, Dakewe Biotech Company Ltd., Shenzhen, China).

### Flow Cytometry Analysis of T Cell Function

#### Intracellular Cytokine Staining

Multiparameter flow cytometry was performed according to a standard protocol (36, 37). For surface staining, single cell suspensions from spleen were prepared at the indicated time points. Freshly isolated lymphocytes were stimulated with ESAT6 (1.25 μg/ml), CFP10 (1.25 μg/ml), and HspX (2.5 μg/ml) for 4 h and subsequently incubated for 8 h with BD GolgiPlug (containing brefeldin A) at 37°C. Samples were stained with anti-CD4-FITC (RM4-5, eBioscience) and anti-CD8-PerCP-Cy5.5 (53–6.7, eBioscience). Then, cells were permeabilized using the BD Cytofix/Cytoperm kit according to the manufacturer's instructions and stained with anti-IFN-γ-APC (XMG1.2, eBioscience). The cell suspensions were analyzed by NovoCyte flow cytometer (ACEC Biosciences, Inc., Zhejiang, China).

#### The Detection of Antigen-Specific CD8^+^ T Cells

CD8^+^ T cells recognizing epitope 4–12 of MTB10.4 were analyzed by flow cytometry. As antigen specific memory T cells are too scanty to be detected directly, the immunized mice were stimulated with BCG (for MH plus LT70 immunization) or MH plus ESAT6 and CFP10 (for MH plus ESAT6 and CFP10 immunization) 3 days before the immunodetection to induce memory T cells proliferating and converting into effector T cells (T_eff_) or effector memory T cells (T_EM_). Cells were stained with MTB10.4_4−12_ PE-conjugated Pro5® Pentamer (ProImmune, Magdalen, United Kingdom) and incubated at 22°C for 10 min. Subsequently, surface staining of these cells was performed using the following anti-mouse monoclonal antibodies including APC-conjugated anti-CD3 (SK7, eBioscience), PerCP-Cy5.5-conjugated anti-CD8 (53–6.7, eBioscience) at 4°C for 30 min. Then, cells were resuspended in PBS and analyzed using NovoCyte flow cytometer.

#### Detection of PD-1 Expression

Cells were resuspended in staining buffer (PBS containing 5% FBS) and blocked with mouse IgG. Subsequently, the anti-mouse monoclonal antibodies including APC-conjugated anti-CD4 (GK1.5), PE-conjugated anti-CD8 (SK1), FITC-conjugated anti-PD-1 (J43), mouse IgG1 isotype control, were added and incubated with cell suspensions at 4°C for 30 min. Cells were then resuspended in PBS and analyzed using LSR Fortessa flow cytometer (BD, NJ, USA). Results are calculated as percent positive cells as indicated.

### BCG Challenge, Bacterial Load Detection, and Survival Rate

#### BCG Challenge and Bacterial Load Detection

To detect the immune protective capability against mycobacterial infection in persistent antigen stimulated mice, the mice constantly stimulated with MH plus LT70 were challenged with 1 × 10^7^ CFU of BCG per mice via caudal vein 6 weeks after the last antigen stimulation or 1 week after IL-2 treatment. Enumeration of CFU in the lungs was determined by serial 10-fold dilutions of whole-organ homogenates on 7H11 medium (BD, NJ, USA) at 3 weeks after infection. Bacterial load is presented as log_10_ CFU ± SEM.

#### BCG Challenge and Survival Rate Detection

Mice constantly stimulated with MH and LT70 were challenged with 1 × 10^9^ CFU of BCG per mice via caudal vein 6 weeks after the last antigens stimulation or one week after IL-2 treatment. The mice without antigen stimulation were used as a naïve control. The survival rates throughout the 90 days were analyzed.

### Statistical Methods

All values are expressed as mean ± SEM. Differences between the variance were analyzed by one-way ANOVA using SPSS13.0 software. A value of *p* < 0.05 was considered statistically significant.

## Results

### Persistent *M. tuberculosis* Antigen Stimulation Impaired Cytokine Production by T Cells

**Figure 2:**
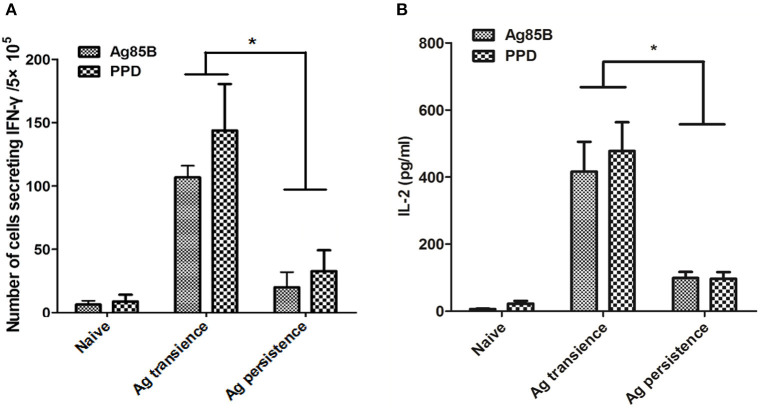
Persistent *M. tuberculosis* antigen stimulation impaired cytokine production. The C57BL/6 mice were primed with BCG once and boosted with *M. tuberculosis* antigens MH plus LT70 (ML) repeatedly for more than 10 weeks. One or three weeks after the last stimulation, mice were euthanized to detect the production of cytokine. **(A)** The numbers of CD4^+^ and CD8^+^ T lymphocytes secreting IFN-γ from persistent ML stimulated mice. **(B)** The concentration of IL-2 produced by T lymphocytes from persistent ML stimulated mice. Mean ± SEM, *n* = 4–6, *p < 0.05.

The C57BL/6 mice were primed with BCG once and boosted with *M. tuberculosis* antigens MH plus LT70 or MH plus ESAT6 and CFP10 repeatedly for more than 10 weeks. One or three weeks after the last stimulation, mice were euthanized to detect the antigen specific immune responses (Figure 1). As for MH plus LT70 stimulation group, after persistent stimulation for 10 weeks, ELISPOT assay showed that the number of CD4^+^ and CD8^+^ T cells producing IFN-γ declined obviously following the stimulation with Ag85B and PPD *in vitro* compared with that of transient antigen stimulation group (Figure 2A). Meanwhile, following Ag85B and PPD stimulation *in vitro*, the quantity of IL-2 secretion from the spleen lymphocytes of persistent stimulation group also decreased significantly than that from the transient antigen stimulation mice (Figure 2B). As for MH plus ESAT6 and CFP10 stimulation group, intracellular cytokine analysis by flow cytometry showed that the frequency of IFN-γ producing CD4^+^ T cells among spleen lymphocytes declined clearly after persistent stimulation for 22 weeks (0.26 ± 0.16) compared with transient antigen stimulation (0.63 ± 0.36; *p* < 0.05), although there were no obvious changes observed comparing transient antigen stimulation (0.55 ± 0.25) with persistent stimulation for 12 weeks (0.78 ± 0.29; data not shown). In these two models, it was confirmed that persistent stimulation by either of the two combinations of antigens caused a decrease in the production of IFN-γ and IL-2.

### Persistent *M. tuberculosis* Antigen Stimulation Decreased the Number of Antigen Specific CD8^+^ T Cells

**Figure 3:**
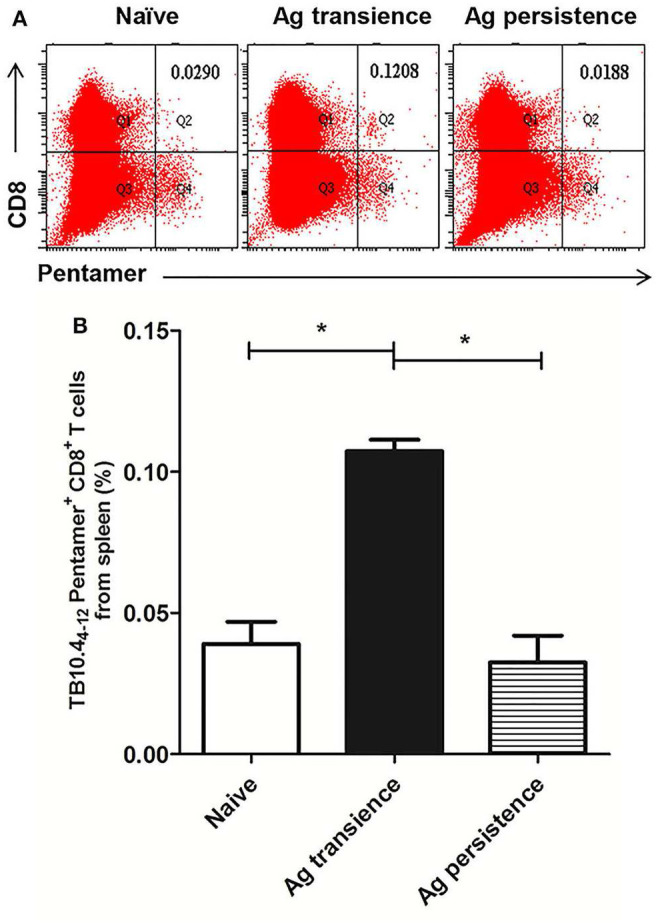
Persistent *M. tuberculosis* antigen MH plus LT70 (ML) stimulation decreased the number of TB10.4_4−12_ pentamer-positive CD8^+^ T cells. The antigen immunized mice were stimulated with BCG 3 days before the immunodetection point. Spleen lymphocytes were isolated and the numbers of TB10.4 pentamer-positive CD8^+^ T cells were detected. **(A)** The flow cytometric analysis of TB10.4_4−12_ pentamer specific spleen CD8^+^ T cells from persistent ML stimulated mice, a representative experiment. **(B)** The percentage of TB10.4_4−12_ pentamer-positive T cells among spleen CD8^+^ T lymphocytes from persistent ML stimulated mice, mean ± SEM. *n* = 4–6, *p < 0.05.

**Figure 4:**
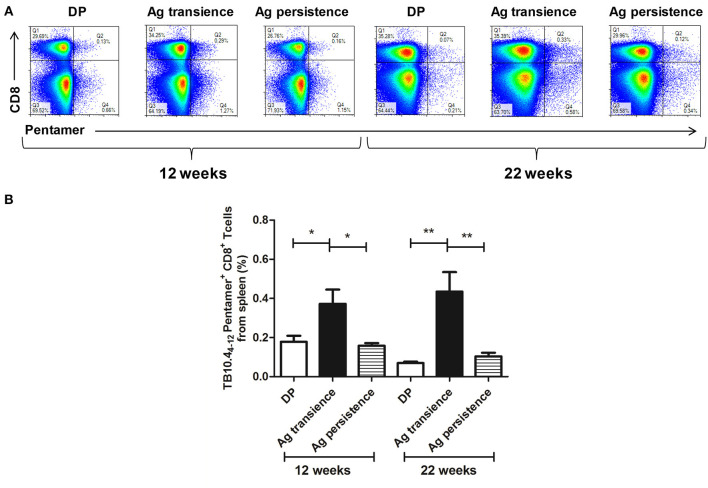
Persistent *M. tuberculosis* antigen MH plus ESAT6 and CFP10 (MEC) stimulation decreased the number of TB10.4_4−12_ pentamer-positive CD8^+^ T cells. The antigen immunized mice were stimulated with MEC 3 days before the immunodetection point. Spleen lymphocytes were isolated and the numbers of TB10.4 pentamer-positive CD8^+^ T cells were detected. **(A)** The flow cytometric analysis of the TB10.4_4−12_ pentamer-positive CD8^+^ T cells from persistent MEC stimulated mice, a representative experiment. **(B)** The percentage of TB10.4_4−12_ pentamer-positive CD8^+^ T cells from persistent MEC stimulated mice, mean ± SEM. *n* = 4–6, *p < 0.05, **p < 0.01.

To observe whether persistent antigen stimulation impair CD8^+^ T cells, the number of antigen specific CD8^+^ memory T cells was analyzed using the method as previously reported (37). In the first experiment, TB10.4_4−12_ specific memory CD8^+^ T cells were formed in the MH plus LT70 transient stimulation mice. However, following MH plus LT70 persistent stimulation for 10 weeks, the numbers of TB10.4_4−12_ specific CD8^+^ T cells (0.03 ± 0.02) decreased clearly in comparison to transient antigen stimulation group (0.11 ± 0.01; *p* < 0.05; Figure 3), which means the memory T cells decreased. The same phenomenon was observed in the second experiment: comparing with MH plus ESAT6 and CFP10 transient stimulation, the numbers of TB10.4_4−12_ specific CD8^+^ T cells decreased after MH plus ESAT6 and CFP10 persistent stimulation for 12 weeks (*p* < 0.05); After stimulation for 10 more weeks, the numbers of TB10.4_4−12_ specific CD8^+^ T cells (0.1 ± 0.04) were significantly lower than antigen transient group (0.44 ± 0.17; *p* < 0.01; Figure 4).

### Persistent *M. tuberculosis* Antigen Stimulation Induced Over-Expression of PD-1 on Spleen CD4^+^ T Cells

**Figure 5:**
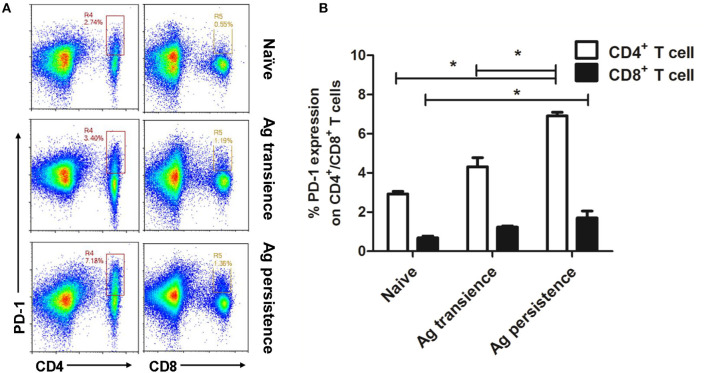
Persistent *M. tuberculosis* antigen stimulation induced overexpression of PD-1 on spleen CD4^+^ T cells. Single cell suspensions from the spleen were resuspended in PBS and the anti-mouse monoclonal antibodies were added and incubated together at 4°C for 30 min. Then, cells were analyzed using flow cytometer. **(A)** The flow cytometric analysis of PD-1 on spleen CD4^+^ and CD8^+^ T cells from persistent MH plus LT70 (ML) stimulated mice, a representative experiment. **(B)** The percentage of PD-1 on CD4^+^ and CD8^+^ T cells from persistent ML stimulated mice, mean ± SEM. *n* = 3, *p < 0.05.

Up-regulation of PD-1 is considered as an indicator of exhausted T cells (3, 38). After persistent MH plus LT70 stimulation for 10 weeks, the percentage of PD-1 positive CD4^+^ and CD8^+^ T cells from spleen lymphocytes were analyzed. The results showed that the expression of inhibitory receptor PD-1 on CD4^+^ T cells (6.91 ± 0.26) increased significantly at late stage of persistent antigen stimulation (*p* < 0.05) than both antigen transient group (4.32 ± 0.65) and naive group (2.93 ± 0.17), while the percentage of increasing PD-1 on CD4^+^ T cells was more obvious than that on CD8^+^ T cells. The expression of PD-1 on CD8^+^ T cells in persistent antigen stimulation group (1.7 ± 0.50) was significantly higher than that in naive group (0.69 ± 0.13; *p* < 0.05; Figure 5).

### Persistent *M. tuberculosis* Antigen Stimulation Reduced the Protection Against BCG Challenge

**Figure 6:**
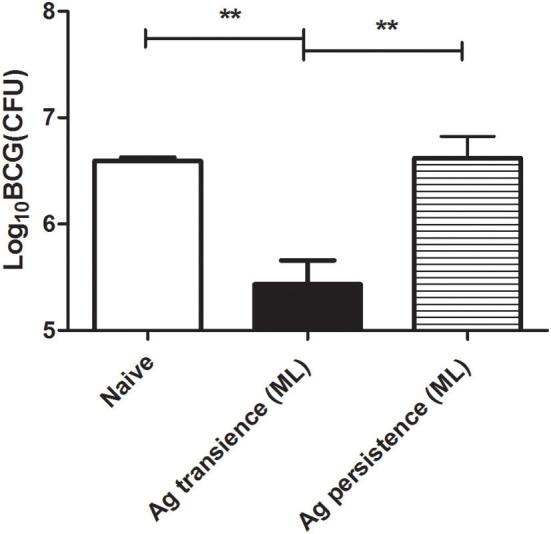
Persistent *M. tuberculosis* antigen stimulation reduced the protection against BCG challenge. To investigate the protective efficacy of the dysfunctional T cells, the MH plus LT70 (ML) stimulated mice were challenged with 1 × 10^7^ CFU of BCG per mice, bacterial load in the lungs was determined at 3 weeks after challenge. Mean ± SEM, *n* = 4, **p < 0.01.

The above results showed that persistent antigen stimulation increased PD-1 expression, impaired the production of cytokine IL-2 and IFN-γ, and reduced the number of memory T cells. To investigate the protective efficacy of the functionally exhausted T cells, the MH plus LT70–stimulated mice were challenged with BCG, then the bacterial load was measured. BCG is an attenuated tubercle bacillus and has weakened virulence. When BCG was used to infect mice, it still could induce the death of mice. The results showed that the bacteria load in the group of persistent antigen stimulation (6.62 ± 0.35 Log_10_ CFU) was significantly higher compared with the group of transient antigen stimulation (5.43 ± 0.39 Log_10_ CFU; *p* < 0.01; Figure 6), indicating that the immune impairment led to the loss of protection against bacterial infection in the persistent antigen stimulation group.

### IL-2 Treatment Restored T-Cell Dysfunction

To investigate whether IL-2 could restore T cell dysfunction, the persistent antigen stimulated mice were treated with IL-2. The function of T cells was analyzed. The results showed that the T cells from the mice persistently stimulated with antigens, either MH plus LT70 or MH plus ESAT6 and CFP10, became functionally restored following IL-2 treatment.

#### The Recovery of IFN-γ and IL-2 Production and Number of Antigen Specific CD8^+^ T Cells

**Figure 7:**
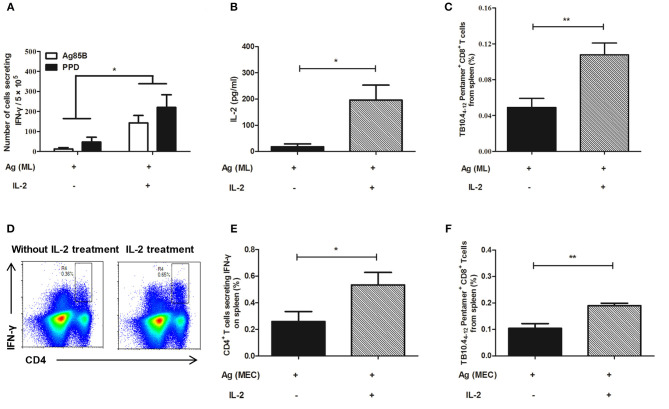
IL-2 treatment restored cytokine production and the number of TB10.4_4−12_ pentamer-positive CD8^+^ T cells in the mouse model. Following persistent antigen stimulation and IL-2 treatment, the cytokine production, and the number of memory T cells were evaluated. **(A)** The number of CD4^+^ T cells secreting IFN-γ from persistent MH plus LT70 (ML)–stimulated mice *in vitro*. **(B)** The level of IL-2 produced by spleen T lymphocytes from persistent ML–stimulated mice. **(C)** The percentage of TB10.4_4−12_ pentamer-positive T cells among spleen CD8^+^ T lymphocytes from persistent ML–stimulated mice. **(D)** The flow cytometric analysis of intracellular IFN-γ produced by spleen CD4^+^ T lymphocytes from persistent *M. tuberculosis* antigens MH plus ESAT6 and CFP10 (MEC)–stimulated mice, a representative experiment. **(E)** The percentage of intracellular IFN-γ production by spleen CD4^+^ T lymphocytes from persistent MEC–stimulated mice. **(F)** The percentage of TB10.4_4−12_ pentamer-positive T cells among spleen CD8^+^ T lymphocytes from persistent MEC–stimulated mice. Mean ± SEM, *n* = 4–6, *p < 0.05, **p < 0.01.

As for persistent MH plus LT70 stimulation, ELISPOT assay showed that compared with persistent antigen stimulated mice, IL-2 treatment increased the fraction of the T cells producing IFN-γ (Figure 7A). Meanwhile, ELISA detection showed that the production of antigen specific IL-2 also increased (*p* < 0.05; Figure 7B). Consistently, flow cytometry analysis showed that the numbers of TB10.4_4−12_ specific CD8^+^ T cells increased significantly from 0.05 ± 0.02 to 0.11 ± 0.02 (*p* < 0.01; Figure 7C), which indicated that the number of memory CD8^+^ T cells increased following IL-2 treatment. Similarly, as for persistent MH plus ESAT6 and CFP10 stimulation, flow cytometry analysis showed that IL-2 treatment improved the percentage of spleen CD4^+^ T cells producing IFN-γ following specific antigen stimulation from 0.26 ± 0.16 to 0.53 ± 0.19 (*p* < 0.05; Figures 7D,E). Meanwhile the numbers of TB10.4_4−12_ specific CD8^+^ T cells increased significantly from 0.1 ± 0.04 to 0.19 ± 0.02 (*p* < 0.01) (Figure 7F).

#### The Change on the Expression of PD-1 on CD4^+^ T Cells

**Figure 8:**
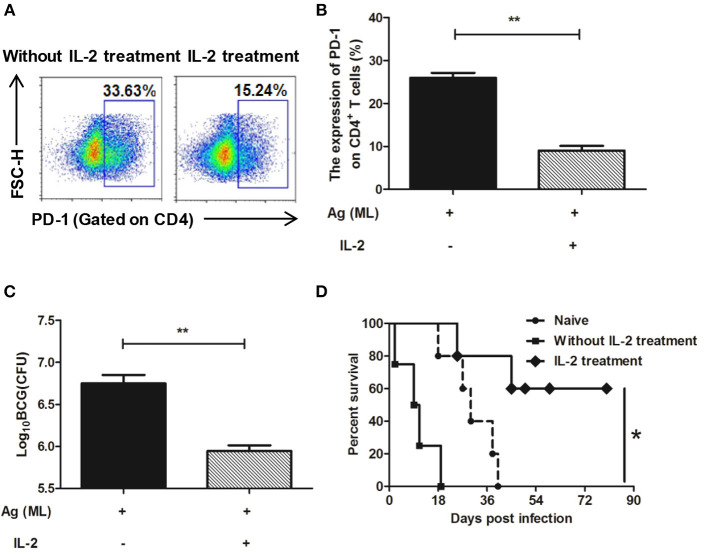
IL-2 treatment decreased the expression of PD-1 on spleen CD4^+^ T cells and restored the immune protection against BCG challenge. Persistent MH plus LT70 (ML) stimulated mice were treated with IL-2, the expression of PD-1 on spleen CD4^+^ T cells and the immune protection against BCG challenge were analyzed. **(A)** The flow cytometric analysis of PD-1 on spleen CD4^+^ T cells from persistent ML–stimulated mice with/without IL-2 treatment, a representative experiment. **(B)** The expression of PD-1 on spleen CD4^+^ T cells from persistent ML–stimulated mice with/without IL-2 treatment. **(C)** The protective efficacy against BCG challenge with/without IL-2 treatment. **(D)** The survival rates of mice after high dose of BCG challenge with/without IL-2 treatment. Mean ± SEM*, n* = 4–5, *p < 0.05, **p < 0.01.

After IL-2 treatment, the expression of PD-1 on CD4^+^ T cells was detected again by flow cytometer. The results showed that in persistent MH plus LT70–stimulated mice, the percentage of CD4^+^ T cells expressing PD-1 was 25.93 ± 1.66, comparing that of 9.05 ± 1.5 in IL-2 treatment group, which indicated that the expression of PD-1 on CD4^+^ T cells decreased following IL-2 treatment (*p* < 0.01; Figures 8A,B).

#### Protective Efficacy Assay and Survival Rate Evaluation

The protective efficacy against BCG challenge after IL-2 treatment was detected. There was a dramatic decrease in the bacterial load in the IL-2 treatment group (5.95 ± 0.11 Log_10_ CFU) than persistent antigen group without IL-2 treatment with T cell dysfunction (6.75 ± 0.18 Log_10_ CFU; *p* < 0.01; Figure 8C). Furthermore, mice were challenged with high dose of BCG and the survival rates throughout the 90 days were analyzed. The data showed that the survival rates of IL-2 treatment group were higher than persistent antigen group without IL-2 treatment (*p* < 0.5; Figure 8D), which indicated that the immune protection recovered following IL-2 treatment. Taken together, our results show that complementing of IL-2 could restore the T cell dysfunction caused by continuous exposure to *M. tuberculosis* antigens.

## Discussion

We hypothesize that continuous antigen stimulation might be the direct reason causing immune dysfunction in TB. To examine the hypothesis, C57BL/6 mice were persistently stimulated with *M. tuberculosis* antigens MH plus LT70 or MH plus ESAT6 and CFP10 and the immune responses were analyzed. The results showed that following persistent antigen stimulation the number of memory T cells was reduced, and the production of antigen specific cytokines IFN-γ and IL-2 decreased. Meanwhile, the expression of T cell-surface inhibitory receptor PD-1 (38) on CD4^+^ T cells increased. All these led to a decrease of protective efficacy against BCG challenge. Considering the possible effect of adjuvant DDA and Poly I:C, the adjuvant was used as control. Repeated stimulation with adjuvant DDA and Poly I:C did not induce immune dysfunction. Our study confirmed that *M. tuberculosis* persistent antigen stimulation induced T cell dysfunction in mice.

Accumulating data show that T-cell exhaustion developed in late state of chronic infections of bacteria and viruses such as LCMV, HBV, HCV, HIV (39–42), and *M. avium* (43). These chronic infections were characterized as high viral loads or persistent bacterial infection. Persistently exposed to antigen stimulation, T cells were constantly activated, and the memory T cells would constantly be converted into terminal T cells. At last the formation and function of T cells would be impaired. Our result gets the support from a recent finding that antigen availability determines the differentiation and function of antigen specific T cells during *M. tuberculosis* infection. They found that due to ESAT-6 was constantly produced throughout *M. tuberculosis* infection, ESAT-6-specific T cells were driven toward terminal differentiation and got functionally exhausted at late stage of infection (44). Our studies also recognized that in some severe TB, such as sputum positive cavitary tuberculosis, persistent stimulation by a large number of bacteria could cause T-cell exhaustion (5).

In this study, persistent stimulation with MH plus LT70, or MH plus ESAT6 and CFP10 induced T-cell dysfunction. The functionally exhausted T cells lose their function of producing cytokines. At the same time, the inhibitory receptor PD-1 on the surface of CD4^+^ T cells was expressed increasingly. Consequently, dysfunctional T cells are unable to clear the pathogens and provide effective protection. In our lab, T-cell dysfunction was also proved to occur in C57BL/6 mice primed with BCG once and boosted with *Mycobacterium bovis* BCG PPD weekly for 16 weeks (45). It was reported that persistent *M. tuberculosis* infection induced CD4^+^ T cell functional exhaustion in animal experiment (3). During chronic *M. tuberculosis* infection antigen-specific T cells expressed high level of PD-1 and had limited capacity to produce IL-2 (46). Our research showed that persistent stimulation with *M. tuberculosis* antigens induced T cell dysfunction, which provides a simple animal model of T cell dysfunction of TB. Besides protein antigens, other antigens in mycobacteria, such as lipid antigens (47), may also induce T cell dysfunction, which remains to be confirmed in further studies.

Among the antigens applied in this study, ESAT6 has strong immunogenicity and was found to be produced along chronic infection, which drove antigen-specific T cells toward terminal differentiation and functional exhaustion at last (44). CFP10 and Mtb10.4 belong to same family of ESAT6 and have the same characteristic as ESAT6. HspX is a main antigen highly expressed in chronic infection (48). Mtb10.4 and HspX were linked together to construct the fusion protein MH, which still kept the immunogenicity of these two antigens (30). The fusion protein LT70 is composed of four main protective antigens (ESAT6, Ag85B, Mtb8.4, and Rv2626c) and has stronger immunogenicity than single antigens (31). It took 10 weeks for LT70 plus MH persistent stimulation to induce T-cell dysfunction, which was 12 weeks shorter than the time for MH plus ESAT6 and CFP10 to induce dysfunction. The main reason could be that LT70 had stronger immunogenicity than single antigens including ESAT6 (31). Therefore, we supposed that T-cell dysfunction induced by persistent strong immune responses, and the occurrence time of T-cell dysfunction might depend on the immunogenicity of antigens. In this experiment applying the antigen combination of MH plus ESAT6 and CFP10, we could observe the process of T cell dysfunction: First, the lose of proliferation of memory CD8^+^ T cells and IL-2 secretion; Then the lose of IFN -γ production.

It is important to find that IL-2 could restore the T cell dysfunction induced by persistent antigen stimulation in mouse model. IL-2 was proven to be effective to reverse CD8^+^ T cell exhaustion in malignant pleural effusion of lung cancer (38). Recombinant IL-2 could enhance T cell survival (49), reverse T cell anergy, and maintain virus-specific CD8^+^ T cell numbers and function during persistent viral infection (26), such as HIV (50, 51), simian immunodeficiency virus (SIV) (52, 53), and LCMV (49). Combining IL-2 therapy with PD-L1 blockade was proved to be more effective in reversing CD8^+^ T cell exhaustion than PD-L1 blockade alone during chronic infections (24). Moreover, IL-2 plays a therapeutic role in MDR-TB (27). Combining with anti-bacterial drugs, IL-2 treatment against MDR-TB led to a higher rate of sputum conversion compared to only drug-treatment controls (54–56). IL-2 could restore T-cell anergy in patients with pulmonary tuberculosis (57). In this study, following persistent MH plus ESAT6 and CFP10 stimulation, IL-2 was used to treat mice daily for 20 weeks, which was designed to mimic IL-2 treatment for chronic TB in clinic. Our results showed that the functionally exhausted T cells could be restored by complementing IL-2: the production of IL-2 and IFN-γ in CD4^+^ T cells increased, the expression of PD-1 on CD4^+^ T cells decreased, the immune protection against BCG challenge improved. It suggests that IL-2 could reverse T cell dysfunction induced by persistent *M. tuberculosis* infection such as in MDR-TB. This explains the effectiveness of IL-2 against MDR-TB and promotes developing novel strategy for intervention against severe TB. Certainly, IL-2 plays a complex role during this process. IL-2 could play an adjuvant role during *M. tuberculosis* antigen stimulation. Moreover, IL-2 might contribute to the hematopoiesis and T cell development in bone marrow. Our recent work showed that IL-2 treatment helps to balance the cytokines homeostasis in bone marrow and recover the dysfunctional hematopoiesis impaired by persistent antigen stimulation (58).

## Conclusion

Persistent *M. tuberculosis* antigen stimulation could lead to T cell dysfunction and supplementing IL-2 contributed to restoring the T cell dysfunction and the exhausted immunity.

## Data Availability Statement

All datasets generated for this study are included in the manuscript/supplementary files.

## Ethics Statement

All animal experiments were carried out under the guidelines of Council on Animal Care and Use, and the protocols were reviewed and approved by Institutional Animal Care and Use Committee of Lanzhou University. Animals were monitored daily and received free access to water and food throughout the study.

## Author Contributions

XL, FL, LM, HN, LP, CG, and XM performed experiments. BZ designed experiments. FL, XL, JC, YZ, and BZ wrote and revised the manuscript.

### Conflict of Interest

The authors declare that the research was conducted in the absence of any commercial or financial relationships that could be construed as a potential conflict of interest.
