# Corrigendum: IL-2 Restores T-Cell Dysfunction Induced by Persistent *Mycobacterium tuberculosis* Antigen Stimulation

**DOI:** 10.3389/fimmu.2020.01671

**Published:** 2020-07-31

**Authors:** Xun Liu, Fei Li, Hongxia Niu, Lan Ma, Jianzhu Chen, Ying Zhang, Liang Peng, Chao Gan, Xingming Ma, Bingdong Zhu

**Affiliations:** ^1^Gansu Provincial Key Laboratory of Evidence Based Medicine and Clinical Translation and Lanzhou Center for Tuberculosis Research, School of Basic Medical Sciences, Lanzhou University, Lanzhou, China; ^2^School of Basic Medical Sciences, Institute of Pathogen Biology, Lanzhou University, Lanzhou, China; ^3^Department of Biology, Koch Institute for Integrative Cancer Research, Massachusetts Institute of Technology, Cambridge, MA, United States; ^4^Department of Molecular Microbiology and Immunology, Bloomberg School of Public Health, Johns Hopkins University, Baltimore, MD, United States; ^5^Center of Life Science, School of Life Sciences, Lanzhou University, Lanzhou, China

**Keywords:** *Mycobacterium tuberculosis*, T cell exhaustion, T cell dysfunction, antigen persistence, IL-2

In the original article, there was a mistake in Figure 3 as published. In Figure 3A some panels were accidentally duplicated. The corrected Figure 3 appears below.

**Figure 3:**
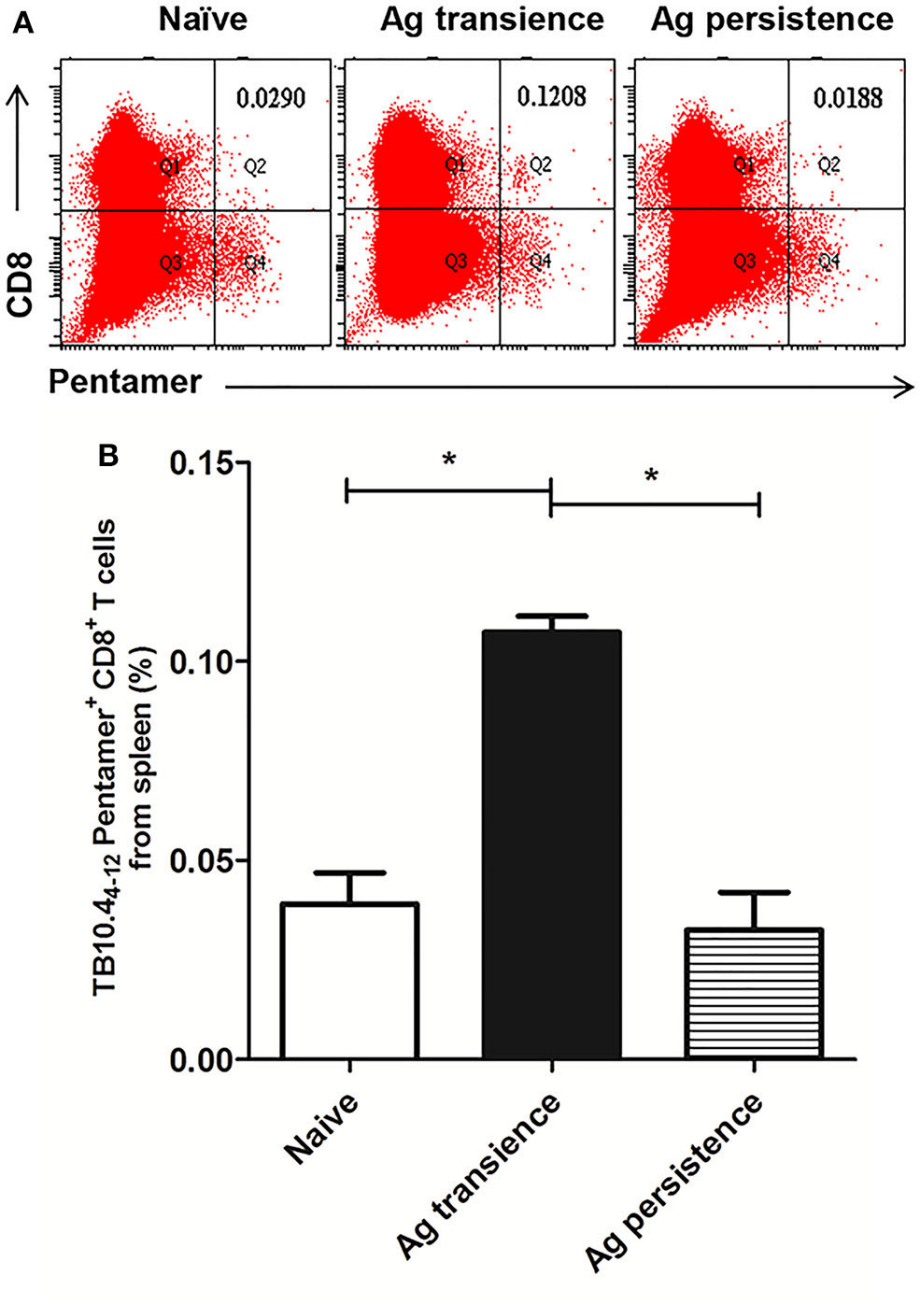
Persistent *M. tuberculosis* antigen MH plus LT70 (ML) stimulation decreased the number of TB10.4_4−12_ pentamer-positive CD8^+^ T cells. The antigen immunized mice were stimulated with BCG 3 days before the immunodetection point. Spleen lymphocytes were isolated and the numbers of TB10.4 pentamer-positive CD8^+^ T cells were detected. **(A)** The flow cytometric analysis of TB10.4_4−12_ pentamer specific spleen CD8^+^ T cells from persistent ML stimulated mice, a representative experiment. **(B)** The percentage of TB10.4_4−12_ pentamer-positive T cells among spleen CD8^+^ T lymphocytes from persistent ML stimulated mice, mean ± SEM. *n* = 4–6, *p < 0.05.

The authors apologize for this error and state that this does not change the scientific conclusions of the article in any way. The original article has been updated.

